# Case Report: A rare case of familial lung cancer requiring pneumonectomy in three male siblings

**DOI:** 10.3389/fonc.2022.947210

**Published:** 2022-08-02

**Authors:** Andrey Kaprin, Oleg Pikin, Andrey Ryabov, Oleg Aleksandrov, Evgeniy Toneev, Ludmila Lubchenko, Ekaterina Zelenova

**Affiliations:** ^1^ P.Herzen Moscow Oncology Research Institute (MORI), Moscow, Russia; ^2^ Peoples’ Friendship University of Russia, Moscow, Russia; ^3^ Ulyanovsk Oncology Center, Ulyanovsk, Russia

**Keywords:** lung cancer, familial cancer, genome sequencing, pneumonectomy, immunotherapy

## Abstract

Lung cancer is a disease with a unique genetic pattern and is occasionally related to hereditary syndromes such as Lynch, Louis–Bar, and Li–Fraumeni. In some patients, germinal mutations may be discovered in combination with somatic alterations. For instance, Li–Fraumeni syndrome often reveals a mixture of *TP53* and *EGFR* mutations. The development of new target therapies necessitates an extensive search for new pathogenic mutations. In this article, we present a rare case report of lung cancer, requiring a pneumonectomy, in three sibling brothers.

## Introduction

Lung cancer is a major socioeconomic threat to modern society and is recognized to play a significant role in morbidity and mortality, even in the most developed countries (1). One of the best-known risk factors is smoking, along with pollution.

In recent years, DNA profiling of malignant diseases has become increasingly popular and widely used in clinical practice. Investigation of pathological mutations may provide an accurate treatment plan, including target therapy, thus having been a cornerstone in modern oncology. Key mutations for lung cancer are *EGFR*, *BRAF*, and *KRAS* and alterations in *PDL-1*, *ALK*, and *ROS1* expression. The current clinical guidelines covering the appropriate administration of immunotherapy and tyrosine kinase inhibitors were devised with consideration of target gene mutations (2).

Germinal mutations are not as common in lung cancer as they are in other tumors, but they are more regularly accompanied with family history. It may be a part of the Lynch, Louis–Bar, or Li–Fraumeni syndromes (3). Given the connection with the latter, germinal mutations in *p53* and *EGFR* are commonly diagnosed (4, 5). To elaborate an explicit and personalized management plan, it is imperative to consider all available options on an extended tumor board with the participation of a geneticist.

## Case history

Three male patients, sibling brothers, were independently diagnosed with lung cancer in the left lung. Patient A (56 years old, the oldest one), patient B (63 years old), and patient C (58 years old) were assessed in 2008, 2016, and 2018, respectively. Each of them was referred to the Regional Cancer Centre in Ulyanovsk for further diagnosis and management.

### Presentation

All patients were admitted with productive cough, associated with occasional bloody expectoration in patients A and B. They had neither significant comorbidities nor environmental or occupational hazards.

### Objective findings

**Figure 1:**
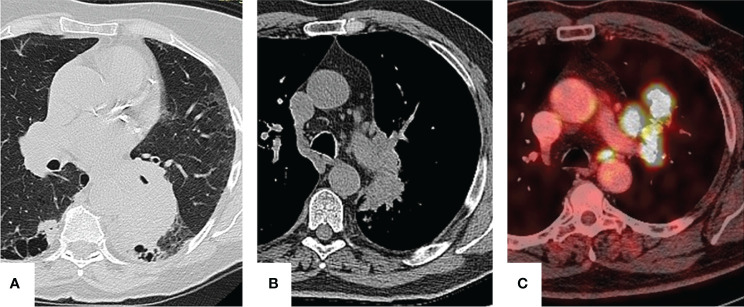
Preoperative imaging. Patient **A**: a 7-cm lesion is seen in the left lower lobe. Patient **B**: perihilar lesion with infiltration of bronchopulmonary lymph nodes. Patient **C**: para-aortic lymph node hypermetabolism.

CT scan showed a peripheral 7-cm lesion of the left lower lobe in patient A, a perihilar lesion of the left upper lobe and a tumor with N1 lymph node involvement in patient B, and a centrally located lesion with hypermetabolism of para-aortic lymph nodes with an SUVmax of 7.34 in patient C (**Figure 1**). In all three cases, a preoperative endoscopic biopsy was performed, which revealed squamous cell carcinoma in patient A and lung adenocarcinoma in patients B and C.

### Diagnosis and management

All patients successfully underwent left-sided pneumonectomy. To downstage the tumor in patient A and because of mediastinal lymph node involvement in patient C, neoadjuvant chemotherapy consisting of etoposide + cisplatin for four cycles was performed.

Postoperative histology showed pT4N0M0, G2, stage IIIa, in patient A; pT2bN1M0, stage IIb, in patient B; and pT2aN2M0, stage IIIa, in patient C. Patients B and C received adjuvant chemotherapy.

After the blood relationship between the patients was revealed, they were referred to a geneticist. Somatic mutations in *EGFR*, *BRAF*, *KRAS*, and *PIK3CA* genes were ordered first and showed no evidence of presence in any of them. Because of serious concerns about familial history, we evaluated the *CHEK2* gene, responsible for the development of different types of cancer, but no mutation was identified. After that, an analysis of microsatellite instability (MSI) was performed, provided by TrueMark MSI Assay (Applied Biosystems, USA) (**Tables 1**, **2**).

**Table 1:** Results of activation mutations and MSI status.

Patient	*EGFR*	*BRAF*	*KRAS*	*PIK3CA*	*CHEK2*	MSI
A	–	–	–	–	Wt	–
B	Wt	Wt	Wt	Wt	Wt	MSI-high
C	Wt	Wt	Wt	Wt	Wt	MSI-high

**Table 2:** MSI status associated with functional alteration of the mismatch repair system in patients B and C.

Locus	Chromosome	Instability
NR27	4q12	Stable
NR21	2q11.1	Unstable for C
NR24	14q11.2	Stable
BAT25	1p12	Stable
BAT26	7q34	Unstable for B and C
CAT25	11q24.2	Stable
BAT40	11q22.2	Unstable for B and C
NR22	1q42.3	Unstable for B and C
ABI-19	1q21.3	Stable
ABI-20B	17p12	Stable
ABI-17	17p13.2	Stable
ABI-16	2p21	Unstable for B and C
ABI-20A	12q24.13	Stable
TH01	11p15.5 (HID)	Match
PentaD	21q22.3 (HID)	Match

Furthermore, in patient A, a complex molecular genetic testing was completed with the usage of a broad genetic panel: *APC*, *ATM*, *AXIN2*, *BARD1*, *BLM*, *BMPR1A*, *BRCA1*, *BRCA2*, *BRIP1*, *CDH1*, *CDKN2A*, *CHEK2*, *DICER1*, *EPCAM*, *GALNT12*, *GREM1*, *MEN1*, *MLH1*, *MLH3*, *MSH2*, *MSH3*, *MSH6*, *MUTYH*, *NBN*, *NF1*, *NTHL1*, *PALB2*, *PMS2*, *POLD1*, *POLE*, *PTCH1*, *PTCH2*, *PTEN*, *RAD51C*, *RAD51D*, *RET*, *SMAD4*, *STK11*, *SUFU*, *TP53*, *TSC1*, *TSC2*, *VHL*, and *WT1*. For the naming of the revealed variations, we utilized the nomenclature from http://varnomen.hgvs.org/.

Data processing was carried out with an automated algorithm, comprised of translation alignment of Genome Reference Consortium Human Build 38 (GRCh38), post-processing of alignment, and detection of variants and quality filter, along with the annotation of revealed variants of all known transcripts for each gene from RefSeq based on the usage of the pathogenic potential of substitution prediction (SIFT, PolyPhen2-HDIV, PolyPhen2-HVAR, MutationTaster, MetaSVM) and evolutionary conservative position calculations by PhyloP and PhastCons.

For an appraisal of population prevalence, we adopted samples from the Genome Aggregation Database (gnomAD), Exome Aggregation Consortium (ExAC), 1000 Genomes, and NHLBI Exome Sequencing Project (ESP6500). The classification of nucleotide sequence was accomplished according to a technical standard of next-generation sequencing (NGS), American College of Medical Genetics and Genomics (ACMG) (**Table 3**).

**Table 3:** Properties of the study.

Total sequences	818,529
Sequence length	2 × 150
Overall nucleotides	116,694,169
Mean coverage	251.8
Revealed variants	467
Variants after pathogenic criteria filtering	1

After amplification and extensive sequencing of lymphocyte’s DNA, we revealed the germinal missense mutation NM_000264.5(PTCH1):c.3941C>T (p.Pro1314Leu, rs1400282737, COSM9550521) in a heterozygous state.

**Figure 2:**
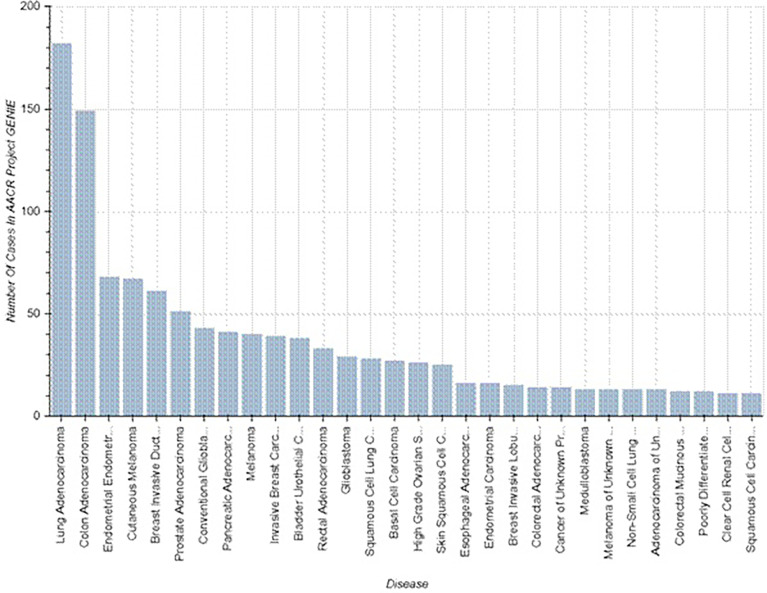
Known cases of malignancy associated with *PTCH1* mutations (https://www.mycancergenome.org/content/alteration/ptch1-mutation/).

The mutation NM_000264.5(PTCH1):c.3941C>T in the gene *PTCH1* is registered in ClinVar and COSMIC databases as a pathologically relevant variant (score 1.00), associated with a high risk of different malignant tumors (**Figure 2**).

## Discussion

According to GLOBOCAN (2021), lung cancer includes 11.6% of all malignant tumors worldwide and is associated with a large amount of 1-year lethality. Despite significant advances in treatment, it is still a remarkably fatal disease with an average rate of 48.4% of those who die within a year since diagnosis (1).

Genetics, environment, and length of affection may influence the possibility and timing of familial lung cancer cases. A meta-analysis, conducted by The International Lung Cancer Consortium (ILCCO) in 2021, revealed a 1.5-fold increased rate of lung cancer in relatives in the first degree (6). The same findings were shown by Cannon-Albright LA (2019) and Loiola de Alencar (2020), which even stated a two-fold increased rate (7, 8). Ang et al. (2020) appraised high-risk factors of lung cancer, such as Asian race compared to non-Asians, age below 50, smoking, and individuals whose two or more relatives are affected (7). This evidence entails a comprehensive study of genetic factors to determine the optimal pathways for diagnosis, management, and subsequent follow-up.

At the present time, over 754 genes are correlated with lung cancer (8). To establish target genes, DNA diagnostics with polymerase chain reaction are widely implicated. More recently, modern approaches such as NGS have been introduced. The technology is used to determine the order of nucleotides in entire genomes or targeted regions of DNA.

The common pathway for DNA analysis in lung cancer is straightforward. In the case of adenocarcinoma (even in dimorphic combination with squamous cell carcinoma), a molecular genetic assay of *EGFR* mutations (18–21 exons), *BRAF*, *V600E*, *ALK*, and *ROS1* is recommended. For negative or unknown results, further investigation must be proceeded with *PDL-1* testing (2).

For a correct perception of the results and to deliberate the familial prepossession, a discussion with a geneticist is advocated. If a connection with one of the genetic syndromes is suspected, a comprehensive DNA testing must be performed.

The *PTCH1* gene encodes the patched homolog 1 protein. PTCH1, a 12-pass transmembrane protein, encompasses two large extracellular loops and two large intracellular loops. The PTCH1 protein is one of the membranous receptors involved in Hedgehog signaling (9). Hedgehog signaling is important for embryonic development and tumorigenesis.

PTCH1 is altered in 2.76% of non-small cell lung carcinoma patients with *PTCH1* mutation present in 2.56% of all non-small cell lung carcinoma patients (10). The available data on lung cancer features associated with *PTCH1* mutation are limited, and no previous study has focused on the long-term survival of these patients. However, it was found that patients with breast cancer and *PTCH1* mutation had more metastasis in the lungs and worse recurrence-free survival (11). The features of lung cancer associated with *PTCH1* mutation remain to be investigated. In view of the poor prognosis in patient A, a regular follow-up should be provided for life.

It is believed that one of the most important universal causes of cancer development is genomic instability. DNA mismatch repair deficiency leads to microsatellite instability and occurs in 15% of colorectal cancers, leading to the ineffectiveness of standard 5-fluorouracil-based chemotherapy (12).

The MSI-H rate in lung adenocarcinoma has been shown to be rare (0.8%) (13). Patients B and C had MSI-high status; therefore, nivolumab or pembrolizumab can be administered in case of progression.

Overall follow-up comprised 11 years for patient A, 5 years for patient B, and 2 years for patient C with no evidence of recurrence. In case of tumor progression, the thorough genetic testing performed in this study may facilitate the selection of an appropriate treatment option.

## Conclusion

This rare observation of familial NSCLC indicates the necessity of a scrupulous analysis of genealogy and elaborate genetic testing. Genetic counseling is mandatory in patients with a familial history of malignancy and the usage of broad panels is advised.

## Data availability statement

The original contributions presented in the study are included in the article/supplementary material. Further inquiries can be directed to the corresponding author.

## Ethics statement

The studies involving human participants were reviewed and approved by the Hertsen Moscow Oncology Research Institute Ethics Committee (#039-B, 23.06.2022). The patients/participants provided their written informed consent to participate in this study. Written informed consent was obtained from the individual(s) for the publication of any potentially identifiable images or data included in this article.

## Author contributions

AK and OP contributed to the conception and design of the study. EZ and LL organized the database. ET and OA wrote the manuscript. All authors contributed to manuscript revision and read and approved the submitted version.

## Conflict of interest

The authors declare that the research was conducted in the absence of any commercial or financial relationships that could be construed as a potential conflict of interest.

## Publisher’s note

All claims expressed in this article are solely those of the authors and do not necessarily represent those of their affiliated organizations, or those of the publisher, the editors and the reviewers. Any product that may be evaluated in this article, or claim that may be made by its manufacturer, is not guaranteed or endorsed by the publisher.
